# Results of a Nationally Representative Seroprevalence Survey of Chikungunya Virus in Bangladesh

**DOI:** 10.1093/infdis/jiae335

**Published:** 2024-06-29

**Authors:** Sam W Allen, Gabriel Ribeiro Dos Santos, Kishor K Paul, Repon Paul, Mohammad Ziaur Rahman, Mohammad Shafiul Alam, Mahmudur Rahman, Hasan Mohammad Al-Amin, Jessica Vanhomwegen, Scott C Weaver, Taylor Smull, Kyu Han Lee, Emily S Gurley, Henrik Salje

**Affiliations:** Department of Genetics, University of Cambridge, Cambridge, United Kingdom; Department of Genetics, University of Cambridge, Cambridge, United Kingdom; Kirby Institute, University of New South Wales, Sydney, Australia; School of Population Health, University of New South Wales, Sydney, New South Wales, Australia; One Health Laboratory, icddr,b, Dhaka, Bangladesh; One Health Laboratory, icddr,b, Dhaka, Bangladesh; Centre for Big Data Research in Health, University of New South Wales, Sydney, New South Wales, Australia; One Health Laboratory, icddr,b, Dhaka, Bangladesh; One Health Laboratory, icddr,b, Dhaka, Bangladesh; Global Health Development, EMPHNET, Dhaka, Bangladesh; One Health Laboratory, icddr,b, Dhaka, Bangladesh; QIMR Berghofer Medical Research Institute, The University of Queensland, Herston, Australia; School of the Environment, The University of Queensland, Herston, Queensland, Australia; Institut Pasteur, Université Paris Cité, Unité Environnement et Risques Infectieux, Cellule d'Intervention Biologique d'Urgence (CIBU), 75015 Paris, France; Department of Microbiology and Immunology, University of Texas Medical Branch, Galveston, Texas, USA; Department of Epidemiology, Johns Hopkins Bloomberg School of Public Health, Maryland, Baltimore, USA; Emory Global Health Institute, Emory University, Atlanta, Georgia, USA; Department of Epidemiology, Johns Hopkins Bloomberg School of Public Health, Maryland, Baltimore, USA; Department of Genetics, University of Cambridge, Cambridge, United Kingdom

**Keywords:** chikungunya, seroprevalence, Bangladesh, epidemiology, arbovirus

## Abstract

There is an increasing global burden from chikungunya virus (CHIKV). Bangladesh reported a major epidemic in 2017, but it was unclear whether there had been prior widespread transmission. We conducted a nationally representative seroprevalence survey in 70 randomly selected communities immediately before the epidemic. We found that 69 of 2938 sampled individuals (2.4%) were seropositive to CHIKV. Seropositivity to dengue virus (adjusted odds ratio, 3.13 [95% confidence interval, 1.86–5.27]), male sex (0.59 [.36–.99]), and community presence of *Aedes aegypti* mosquitoes (1.80 [1.05–3.0]7) were significantly associated with CHIKV seropositivity. Using a spatial prediction model, we estimated that across the country, 4.99 (95% confidence interval, 4.89–5.08) million people had been previously infected. These findings highlight high population susceptibility before the major outbreak and that previous outbreaks must have been spatially isolated.

## Teaser

We use a nationally representative seroprevalence study to estimate the underlying burden of chikungunya before a major outbreak in 2017. We found that the population had very limited immunity at the time, but the vector was widespread.

Chikungunya virus (CHIKV) is an *Aedes (Stegomyia)* mosquito*–*transmitted arbovirus first detected in Tanzania in 1953, which has since spread from its origins in Africa to Asia and South America, as well as sporadic outbreaks elsewhere [1]. The hallmark symptoms of chikungunya are abrupt onset of fever and joint pain [2, 3], commonly accompanied by a rash [4]. The clinical presentation of chikungunya in humans is similar to that of dengue and a range of other illnesses, so it is often misdiagnosed [2, 5–9]. About half of infected individuals experience long-term effects, the most common of which is arthralgia [10–15], which can continue for months or even years after infection, severely decreasing quality of life [16–18]. High attack rates coupled with frequent severe, persistent symptoms means that CHIKV outbreaks can place a large burden on communities, especially in lower-middle-income countries [2].

Limited access to testing and misdiagnoses, often as dengue, mean that entire CHIKV epidemics are frequently missed, especially in lower-middle-income countries [19, 20], and we rarely have a good understanding of the underlying burden from CHIKV in any affected setting, nor individual-, household-, and population-level predictors of infection risk. This critical knowledge gap means we do not know where to appropriately target interventions.

This is becoming increasingly relevant. The first CHIKV vaccine was licensed by the US Food and Drug Administration in November 2023 [21]. In addition, the targeted release of *Wolbachia*-infected mosquitoes can reduce CHIKV incidence [22, 23]. In this context, seroprevalence studies can help, especially as CHIKV infection appears to result in long-lasting and immunizing antibodies [24]. By measuring the presence of antibodies in the population, we can quantify its underlying level of infection history [20]. Furthermore, by combining the results of seroprevalence studies from multiple locations with mathematical models, we can estimate the burden across the population and the changing level of immunity [25].

Here we focus on Bangladesh, where CHIKV was first identified in 2008 during an outbreak in the northwest of the country in 2 villages near the Indian border [26]. Subsequently, localized outbreaks were detected between 2011 and 2016 [2, 27]. In 2017, Bangladesh experienced a far larger outbreak, with infections reported nationwide with as many as a million reported cases in total [2, 28, 29]. During this outbreak, 17 of the 64 districts in the country reported cases, especially around the large urban hubs, but there was likely undetected transmission elsewhere. It remains unclear whether there was substantial transmission across the country before the large outbreak in 2017. In this project, we present the results of a nationally representative seroprevalence study from Bangladesh. We visited communities around the country in 2016, allowing us to quantify the level of transmission, identify risk factors for infection, and determine the level of immunity before the major outbreak.

## METHODS

### Data Collection

The protocol of the study has been described elsewhere [25]. Briefly, to obtain a nationally-representative sample, 70 of the 97 162 communities listed in the 2011 census were selected at random, with the likelihood of selection being proportional to population size. Each community was visited by the study team, who spent ≥5 days within each community. Visits occurred during October 2015 to January 2016. A further visit was made during June and July 2016 to communities where no *Aedes* had been previously trapped, for additional mosquito collection. Communities where *Aedes* were still not found after the second visit were defined as having an absence of *Aedes aegypti* and *Aedes albopictus*. The study team randomly selected ≥10 households from each community. The heads of selected households were informed of the study and invited to participate. If they agreed, all other members of the household aged >6 months were also invited to participate. Data collection was deemed to be complete for a community when ≥40 serum samples were obtained from ≥10 households.

The head of each participating household was led through a household questionnaire with a variety of questions regarding socioeconomic status, such as education level, estimated household income and access to electricity. In addition, this questionnaire asked whether households had used any form of mosquito control in the last week and whether any member of the household owned land away from their home [30].

Each consenting household member (including the household head) was also guided through an individual-level questionnaire. If individuals were too young to answer this by themselves, an older household member was asked to answer on their behalf. These questionnaires covered demographic questions, such as age and sex, and also asked when participants had last travelled outside of the community [30].

All individuals who provided consent also had 5 mL of venous blood withdrawn by a phlebotomist. These blood samples were centrifuged, and serum was then extracted separately and shipped in nitrogen dry shippers to icddr,b (International Centre for Diarrhoeal Disease Research, Bangladesh) laboratories in Dhaka. Individuals who were ill at the time of the survey were excluded from serum sampling. All serum samples were tested for antibodies against chikungunya to identify evidence of prior infection. This was done using a microsphere-based multiplex immunoassay that measured the fluorescence intensity to both the recombinant E2 glycoprotein of the CHIKV and the background level of antibody activity at the individual level using a recombinant human O^6^-methylguanine-DNA methyltransferase protein (SNAP-tag).

### Assay Validation

To determine the optimal cutoff point to determine CHIKV serostatus, we conducted plaque reduction neutralization testing (PRNT) in a subset of 89 samples (testing conducted by the University of Texas Medical Branch). Using the results of PRNT and the Luminex measures, we calculated the receiver operating characteristic curve. This allowed us to estimate the area under the curve and identify the threshold that maximized sensitivity and specificity.

### Regression Analyses

We used the R-INLA package, which applies the integrated nested Laplace approximation (INLA) method, a bayesian approach to statistical inference for gaussian Markov random field models [31]. A key benefit of this method is that it can accommodate a range of these models, including those with a spatial component. R-INLA allows these to be added to the model as random effects. This means that the spatial autocorrelation inherent in epidemiological data can be accounted for to isolate the role of random spatial variation [32]. We modeled the dependence of 2 observations in this distribution using a covariance function, with the Matérn covariance function. R-INLA's default priors were used beyond the setting of the spatial field, where a fixed smoothness parameter of 〈 = 2 was set. This represents a moderately smooth spatial field and is a commonly selected value [33].

Covariates were divided into individual level (age, sex, dengue serostatus, and time since last leaving the community), household level (income, highest educational level achieved by head of household, electricity in home, owning the home, owning land away from the home, and use of mosquito control in the last week), and community level *(Ae. aegypti* captured in the community*, Ae. albopictus* captured in the community, and log population density). First, each covariate was included in a univariate logistic regression using R-INLA to assess individual relationships with serostatus. Random intercepts were also included for both the household and the community to account for correlation of observations within these sites. Following this, all covariates were included in a multivariable analysis. This generated an odds ratio (OR) and 95% confidence interval (CI) for each covariate from both univariate regression and an adjusted OR and 95% CI from multivariable regression.

To explore the importance of the spatial correlation structure and the random household and community intercepts, additional models with different combinations of these included were also built. In total, 6 models were created: (1) the base model, featuring a Matérn spatial correlation structure, a random community intercept, and a random household intercept; (2) a Matérn spatial correlation structure and a random household intercept only; (3) a Matérn spatial correlation structure and a random community intercept only; (4) a Matérn spatial correlation structure only; (5) random household and community intercepts only; and (6) a mixed-effects model, with random household but fixed community intercepts only.

### Household Infection Risk

To investigate whether living with a seropositive individual was a risk factor for being seropositive oneself, the risk ratio for living with a seropositive individual was calculated in the subset of communities with ≥1 seropositive individual. The risk ratio was then calculated as risk ratio = *a*/*b*, where *a* represents the proportion of seropositive individuals living with seropositive individuals and *b*, the proportion of seropositive individuals living with seronegative individuals. This risk ratio was first calculated, and then bootstrapped for 1000 iterations to generate a distribution of estimates, from which a mean and 95% CI were extracted.

### Mapping CHIKV Risk Across Bangladesh

Seroprevalence by community was mapped by community to visualize the general spatial distribution of chikungunya in 2015–2016. Seroprevalence was defined as the number of individuals with detectable anti-CHIKV antibodies, expressed as a proportion of the total number of individuals surveyed in that community, calculated as the number of seropositive individuals divided by the total population; 95% CIs were also calculated for community seroprevalence, using the Clopper-Pearson estimation method, which is based on the exact binomial distribution. Seroprevalence was calculated for each community in the study and mapped to visualize spatial trends.

To explore infection risk across Bangladesh, a grid of 1 × 1-km^2^ cells was placed over the country. A bayesian framework featuring a Matérn spatial correlation structure was used to fit the model. Covariates from the multivariable regression could not be added to the spatial prediction because values for these covariates are not available for areas outside the study sites. Salje et al [25] found that the inclusion of additional covariates (eg, age and sex) beyond the spatial covariance term that can be obtained from demographic data did not markedly improve predictive accuracy, so these were not added to reduce unnecessary model complexity. The model was then fit to the 1 × 1-km^2^ grid across the country to predict the seroprevalence in each of these cells.

To estimate the total number of people ever infected with CHIKV in Bangladesh at the time of the survey, the population density in each cell was multiplied by the fitted seroprevalence in each cell. CIs were generated by taking the 0.025 and 0.975 estimates from the model and applying the same technique.

To test the predictive performance model, a cross validation was performed. One thousand iterations were performed, with 10 of the 70 communities left out during model fitting for each run. The model was then used to predict the seroprevalence in the 10 test communities. The mean predicted seroprevalence for each community was then used to generate an estimated seroprevalence, which was compared with the observed seroprevalence in each of the held-out communities.

### Ethical Clearance

The icddr,b and Centers for Disease Control and Prevention ethical review boards approved this study (protocol no. PR-14058). All participating adults gave written informed consent. Children involved in the study had written informed consent provided on their behalf by parents/guardians.

## RESULTS

In 2016 we visited 70 randomly selected communities in Bangladesh, collected blood and administered questionnaires from 2938 individuals. The individuals were largely representative of the population of Bangladesh, with some underrepresentation in the youngest age group (Supplementary Figure 1). Simultaneously, we collected mosquitoes using BG-Sentinel traps. There was a mean of 42 participating individuals per community (range, 39–57) and a mean of 10 participating households per community (range, 10–12). The mean age of participants was 30 years, and 52% of participants were female. For a subset of samples (n = 89), we compared our Luminex-based antibody values with those of PRNT, estimated an area under the curve of 0.95 and identified 5.5. as the optimal cutoff point (sensitivity and specificity both >95%) (Supplementary Figures 1 and 2).

**Figure 1.**
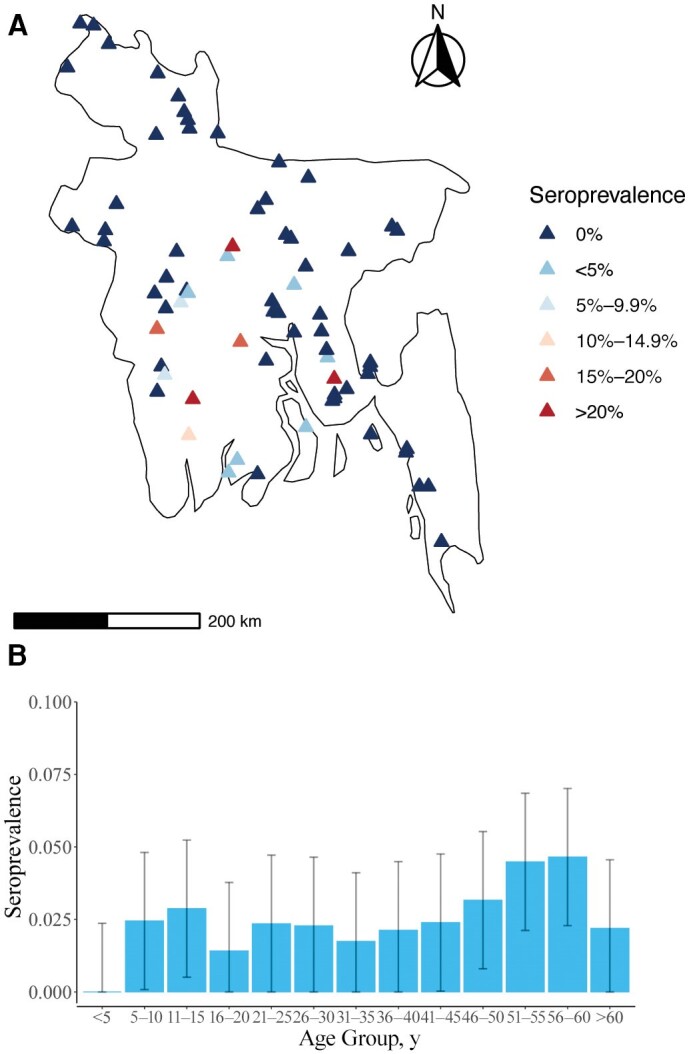
Proportion seropositive. *A*, Map of sampled communities and proportion seropositive to chikungunya virus **(**CHIKV). *B*, Proportion seropositive by age.

Using this threshold to define seropositivity, we found that among the participants, 2.4% were seropositive to CHIKV, with all seropositive individuals coming from 16 communities (23% of all communities) concentrated in the central and south of the country (Figure 1). Among seropositive communities, the mean seropositivity was 10.5%, ranging from 2.1% (95% CI, 0%–2.1%) to 39.0% (27.3%–53.1%). Notably, only a single individual from the 3 communities in Dhaka city was seropositive. Seropositivity was largely consistent across age groups, except for children aged <5 years, who had a seropositivity of 0%, compared with 2.4% for those >5 years old (*P* = .26) (Table 1). We observed greater seropositivity among female versus male participants (2.8% vs 1.8%; *P* = .09). Seropositivity to dengue virus (seen in 24% of the study population), which is transmitted by the same vector, was correlated with chikungunya seropositivity (4.9% vs 1.6%; *P* <.001).

**Table 1.** Individual-, Household- and Community-Level Characteristics of Participants Across Bangladesh, Stratified by Chikungunya Virus Serostatus in 2015–2016

Characteristic	Total No.	Participants, No. (%)^a^		*P* Value^b^
		Seropositive (n = 69)	Seronegative (n = 2869)	
Individual level				
Age, y				
<5	88	0 (0)	88 (100)	NA
5–10	341	7 (2)	334 (98)	
11–20	737	16 (2)	721 (98)	
21–30	518	12 (2)	506 (98)	
31–40	417	8 (2)	409 (98)	
41–50	367	10 (3)	357 (97)	
51–60	242	11 (5)	231 (95)	
>60	228	5 (2)	223 (98)	
Sex				
Female	1532	43 (3)	1489 (97)	.09
Male	1406	26 (2)	1380 (98)	
Dengue status				
Seropositive	697	34 (5)	663 (95)	<.001
Seronegative	2237	35 (5)	2202 (98)	
Unknown	4	0	4	
Time since last leaving community, d				
≤180	1704	41 (2)	1663 (98)	.8
>180	1234	28 (2)	1206 (98)	
Living with someone CHIKV seropositive				
No	2748	30 (1)	2718 (99)	<.001
Yes	190	39 (21)	151 (79)	
Household level				
Annual income, taka (100 taka = $0.9)				
<10 000	850	18 (2)	832 (98)	.8
10 000–20 000	1107	26 (2)	26 (2)	
>20 000	969	25 (3)	25 (3)	
Unknown	12	0	12	
Household head’s education				
None	908	23 (3)	885 (97)	.6
Primary school	759	14 (2)	745 (98)	
High school	797	20 (3)	777 (97)	
Beyond high school	401	12 (3)	389 (97)	
Unknown	73	0	73	
Electricity in home				
No	273	4 (1)	269 (99)	.3
Yes	2665	65 (2)	2600 (98)	
Ownership of home				
No	187	7 (4)	180 (96)	.2
Yes	2751	62 (2)	2689 (98)	
Ownership of land away from home				
No	598	22 (4)	576 (96)	.02
Yes	2340	47 (2)	2293 (98)	
Mosquito control used				
No	1067	21 (2)	1046 (98)	.3
Yes	1871	48 (3)	1823 (97)	
Community level				
*Aedes aegypti* captured				
No	1973	33 (2)	1940 (98)	<.001
Yes	965	36 (4)	929 (96)	
*Aedes albopictus* captured				
No	1707	43 (3)	1664 (97)	.5
Yes	1231	26 (2)	1205 (98)	
Community type				
Rural	2185	47 (2)	2138 (98)	.2
Urban	753	22 (3)	731 (97)	
Division				
Barisal	166	5 (3)	161 (97)	.3
Chittagong	779	18 (2)	761 (98)	
Dhaka	733	20 (3)	713 (97)	
Khulna	334	25 (7)	309 (93)	
Rajshahi	336	1 (1)	335 (99)	
Rangpur	462	0 (0)	462 (100)	
Sylhet	128	0 (0)	128 (100)	

Abbreviations: CHIKV, chikungunya virus; NA, not applicable.

^a^Seropositivity was determined based on the presence of immunoglobulin G antibodies against CHIKV.

^b^Calculated with Pearson χ^2^ test.

Travel history and household-level factors appeared similar across the 2 groups, with the exception of owning land away from the home, which was linked to higher levels of seropositivity. We found that having *Ae. aegypti* captured in the community (23 of 70 communities) was linked to seropositivity (3.7% vs 1.7%; *P* <.01) but having *Ae. albopictus* captured (29 of 70 communities) was not (*P* = .5). We fitted a variogram to the serostatus of individuals as a function of their spatial separation and found spatial dependence in serostatus extended to distances of about 20 km (Supplementary Figure 4).

We used logistic regression to identify covariates associated with seropositivity. We compared models with or without a spatial covariance term, as well as models with or without household- and community-level intercepts. The best-fitting model, as measured by the Watanabe-Akaike information criterion, a measure for model comparison, included random household- and fixed community-level intercepts; however, covariate estimates were largely consistent across the different models considered (Supplementary Table 1 and Supplementary Figure 5). We found that most factors were not associated with CHIKV seropositivity (Table 2). This includes the presence of *Ae. Albopictus* in the community, household income, and travel history. However, age <5 years (adjusted OR, 0.00 [95% CI, .00–.00]), seropositivity for dengue virus (3.13 [1.86–5.27]), male sex (0.59 [.36–.99]), owning land away from the home (0.49 [.28–.87]), population density (0.78 [.61–.99]), and presence of *Ae. aegypti* in the community (1.80 [1.05–3.07]) were significantly associated with serostatus. Either *Ae. aegypti* or *Ae. albopictus* were found in 46 of the communities (66%) (Supplementary Figure 6).

**Table 2.** Results of Univariate and Multivariable Logistic Regression

	Univariate Model	Multivariable Model^a^
Characteristic	OR (95% CI)	Adjusted OR (95% CI)
Individual level		
Age group, y		
<5	0.00 (.00–.00)	0.00 (.00–.00)
5–10	0.93 (.38–2.29)	0.93 (.37–2.30)
11–20	Reference	Reference
21–30	1.08 (.51–2.31)	0.83 (.38–1.80)
31–40	0.87 (.37–2.04)	0.62 (.26–1.49)
41–50	1.27 (.57–2.83)	1.00 (.44–2.26)
51–60	2.21 (1.01–4.85)	1.91 (.85–4.28)
>60	0.95 (.34–2.61)	0.73 (.26–2.08)
Sex		
Female	Reference	Reference
Male	0.65 (.40–1.07)	0.59 (.36–0.99)
Dengue status		
Seronegative	Reference	Reference
Seropositive	3.18 (1.96–5.17)	3.13 (1.86–5.27)
Time since last leaving community, d		
≤180	Reference	Reference
>180	0.96 (.59–1.56)	1.03 (.61–1.76)
Household level		
Annual income, taka (100 taka = $0.9)		
<10 000	Reference	Reference
10 000–20 000	1.10 (.60–2.03)	1.03 (.54–1.99)
>20 000	1.17 (.62–2.19)	1.20 (.59–2.41)
Household head’s education		
None	Reference	Reference
Primary school	0.79 (.40–1.54)	0.82 (.40–1.66)
High school	1.04 (.57–1.91)	1.01 (.52–1.96)
Beyond high school	1.25 (.62–2.54)	1.40 (.65–3.01)
Electricity in home		
No	Reference	Reference
Yes	1.84 (.66–5.09)	1.61 (.54–4.84)
Ownership of home		
No	Reference	Reference
Yes	0.67 (.30–1.49)	0.62 (.23–1.67)
Ownership of land away from home		
No	Reference	Reference
Yes	0.55 (.33–0.92)	0.49 (.28–0.87)
Mosquito control		
No	Reference	Reference
Yes	1.29 (.77–2.18)	1.24 (.71–2.18)
Community level		
*Aedes aegypti* captured		
No	Reference	Reference
Yes	2.25 (1.37–3.68)	1.80 (1.05–3.07)
*Aedes albopictus* captured		
No	Reference	Reference
Yes	0.88 (.53–1.47)	0.98 (.57–1.68)
Population density (log scale)	1.06 (.88–1.27)	0.78 (.61–0.99)

Abbreviations: CI, confidence interval; OR, odds ratio.

^a^The multivariable model selected included random household intercepts and fixed community intercepts but no spatial field (model 6), on the basis of the Watanabe-Akaike information criterion (Supplementary Table 1).

We next explored whether, within the communities where CHIKV seropositivity was detected, living with a seropositive individual was a risk factor for being seropositive. We found that within these communities, individuals who lived with a seropositive householder member had 2.80 (95% CI, 1.47–4.85) times the probability of being seropositive as individuals living with only seronegative individuals.

**Figure 2.**
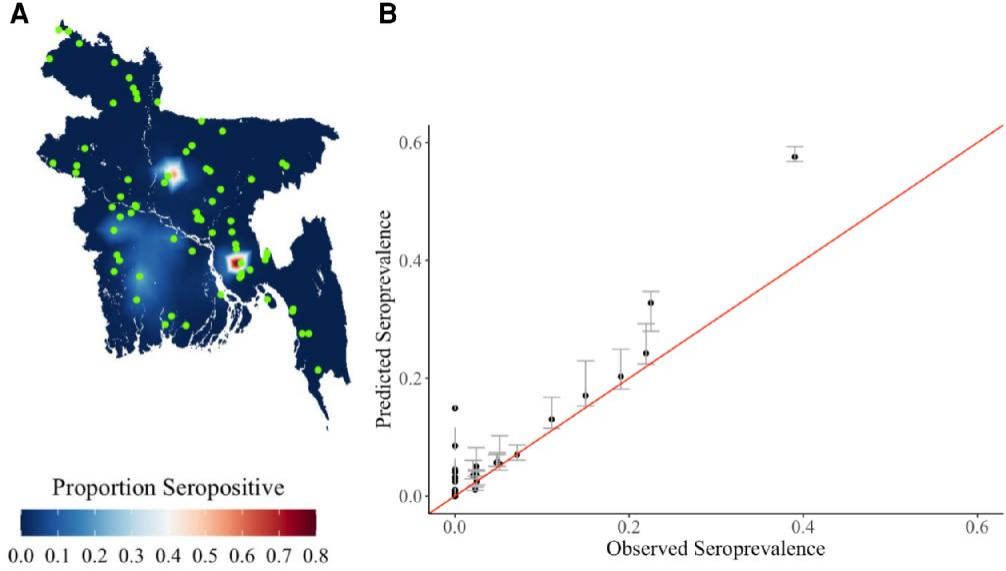
Estimated map of seropositivity. *A*, Modeled seropositivity in Bangladesh in 2016 *B,* Held-out cross-validation, in which communities were removed from the model-fitting process and the rest of the data used to fit models. The plot shows the comparison with the observed versus the predicted in the removed locations.

To estimate the overall seropositivity across the country, we used a spatial prediction model. We used the estimated population distribution in the country to identify the number of infected individuals. Overall, we estimate that 4.99 million people (95% CI, 4.89–5.08 million) in Bangladesh had been infected with CHIKV at some point in their lives as of 2016, with the highest risk concentrated to a few focal hot spots (Figure 2*A*). This equates to about 2.49% (95% CI, 2.45%–2.54%) of the national population, consistent with the estimate produced using the crude proportion seropositive among individuals in the serosurvey (2.35% of the population, or about 4.70 million individuals). To validate our spatial prediction model, we removed all data from a subset of individual communities in turn from our model and used the remaining data to fit a new model. We then used the fitted model to predict in the removed locations. We found we could accurately predict seropositivity in the removed locations (Pearson ρ = 0.95) (Figure 2*B*).

## DISCUSSION

The results from this first nationally-representative serosurvey of CHIKV infection in Bangladesh demonstrate that by 2016 CHIKV had been present in parts of the country, especially the South. Overall, only a relatively small proportion of the population, representing about 5 million individuals, had previously been infected. The high level of population susceptibility at this time can help explain the magnitude and spatial extent of the subsequent major outbreak in 2017.

CHIKV seropositivity was relatively constant across age groups, which indicates that individuals of all ages have had the same cumulative exposure to risk of infection. This is indicative of a recent emergence of CHIKV in Bangladesh, though the decreased seropositivity among those aged <5 years suggests limited exposure in the years immediately preceding the serosurvey. We explored a wide range of individual-, household- and community-level risk factors to identify drivers of infection risk. Seropositivity to dengue virus, another virus transmitted by the same vectors, was an important predictor. This highlights the overlapping risk across *Aedes*-transmitted arboviruses, as previously identified elsewhere [34].

We identified a strong effect of the household, with individuals much more likely to be seropositive if they lived with other seropositive individuals. This finding is consistent with previous findings from Bangladesh and elsewhere that have identified the limited flight range of the vector as driving household infection risk, as biting typically occurs in the peridomestic environment [35]. A strong correlation of serostatus by household has also been observed with dengue virus [36]. Sex was another notable predictor, consistent with results from both Bangladesh and elsewhere that have consistently shown infection risk is higher among females [19, 30, 35, 37, 38]. It has been suggested that differences in mobility patterns may explain this increased risk, with women in Bangladesh spending more time in and around the home where CHIKV vector mosquitoes reside [35].

Our results suggest that, while before 2017 CHIKV outbreaks in Bangladesh have been spatially constrained, there was always the risk of a widespread epidemic. It remains unclear why prior outbreaks in the country died out without spreading widely. We identified either *Ae. aegypti* or *Ae. albopictus* mosquitoes in most communities, suggesting that conditions were suitable for transmission across the country. Introductions may have previously been in rural communities, which are less connected to urban hubs, and potentially died out from entering cooler parts of the year [35]. As population mobility continues to increase, we can expect even wider spread of both *Aedes* vectors and a concurrent increase in arbovirus outbreak risk [39]. In Brazil, which has suffered CHIKV outbreaks annually since introduction of the virus 10 years ago, transmission at a local level is also highly spatially heterogeneous, with many outbreaks being spatially constrained. This pattern has been suggested to be linked to reduced mobility among symptomatic individuals [40, 41].

This project highlights the utility of nationally representative seroprevalence studies, especially when combined with mathematical models. Using a sampling frame of all communities in Bangladesh allows us to generalize to the wider country. These same samples were used to create risk maps for a wide range of other pathogens, including cholera, dengue, and hepatitis E [25, 42, 43]. Furthermore, the increased use of multiplex serology allows the parallel testing of multiple pathogens, maximizing insights from individual blood draws and limiting the need for numerous freeze-thaw cycles.

We note that our modeled estimate of seropositivity at the national level was very consistent with the crude level of seropositivity in our sample set (2.5% vs 2.4%). It is certainly possible that we did not sample communities affected by localized outbreaks, but those outbreaks would not markedly change our estimates for the overall population level immunity for Bangladesh. We note that our out-of-sample validation of the model suggested some overestimation of the seropositivity, which may mean the true seropositivity was even slightly lower than we estimated. Selection bias may have arisen, in that individuals who were away from communities during visits, and hence more likely to travel frequently, may not have been able to participate. However, to minimize this risk, the study team arranged to visit households again when members were expected to return from travel. The travel covariate is also limited in that the questionnaire asked about most recent travel outside the community, which does not provide information on the frequency of, reason for, or destination of travel. All of these could be relevant to CHIKV infection risk. We note that the 2 locations with the highest predicted seroprevalence were overpredicted by the model, suggesting some model misspecification in estimating high attack rates.

In conclusion, we demonstrate high CHIKV susceptibility across Bangladesh before the major outbreak in 2017 and show that prior outbreaks were largely spatially isolated in nature. Given the potential for large outbreaks, Bangladesh should be prioritized for new interventions, such as vaccines and *Wolbachia*-based vector control, as they become available.

## Supplementary Data

Supplementary materials are available at *The Journal of Infectious Diseases* online (http://jid.oxfordjournals.org/). Supplementary materials consist of data provided by the author that are published to benefit the reader. The posted materials are not copyedited. The contents of all supplementary data are the sole responsibility of the authors. Questions or messages regarding errors should be addressed to the author.

## Supplementary Material

jiae335_Supplementary_Data

## Notes

***Acknowledgments***. We thank the staff of the World Reference Center for Emerging Viruses and Arboviruses at the University of Texas Medical Branch for technical assistance.

***Financial support*.** This work was supported by European Research Council (grant 804744), the Centers for Disease Control and Prevention, and the National Institutes of Health (grants R24 AI120942 and U01AI151801).
