# Supplementary material for: Results of a Nationally Representative Seroprevalence Survey of Chikungunya Virus in Bangladesh
Source: J Infect Dis. 2024 Jun 29;230(5):e1031–8. doi: 10.1093/infdis/jiae335 (PMC11565896; doi:10.1093/infdis/jiae335)
Supplement: jiae335_Supplementary_Data [file jiae335_supplementary_data.pdf]

Supplementary Figures and Tables

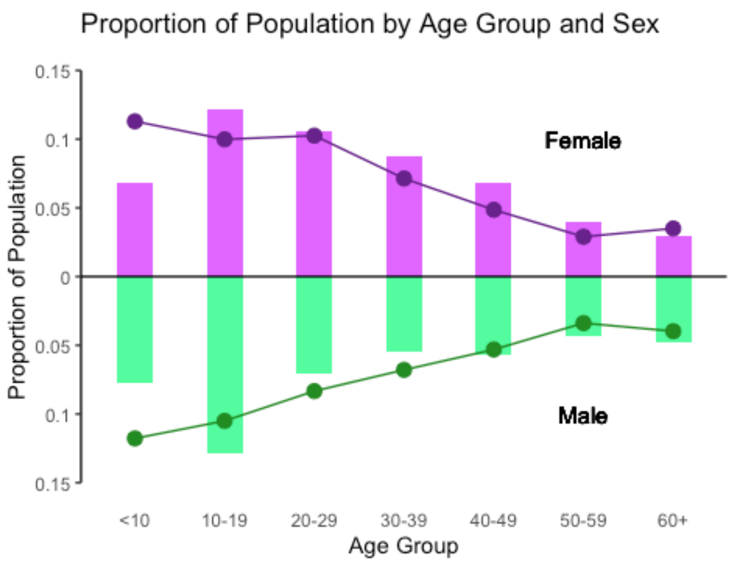

Figure S1: Proportion of population by age and sex, as compared to the national census. Bars represent study participants and lines the 2011 national census.

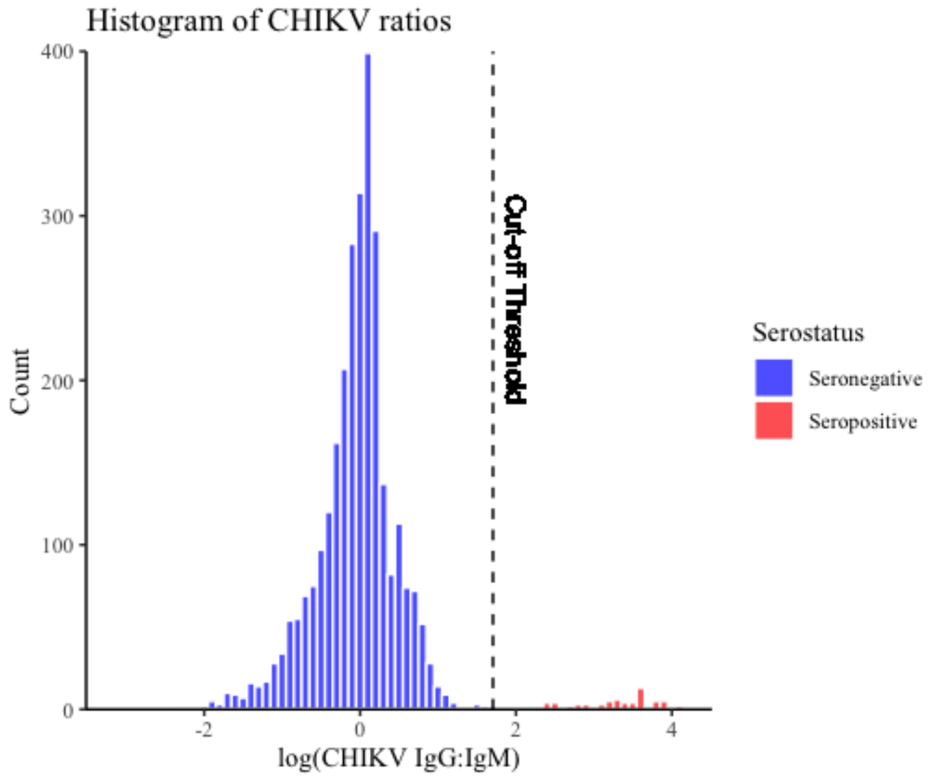

Figure S2. Histogram of the ratios between fluorescence intensity to CHIKV and the control SNAP-tag protein with the cutoff point of 5.5 marked (dashed line). Samples to the left of the dashed line are considered seronegative, and those to the right seropositive.

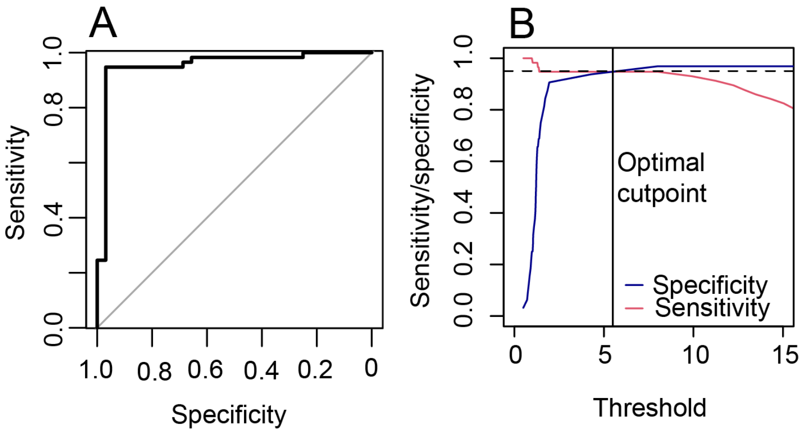

Figure S3: Comparison of PRNT and Luminex data to obtain optimal cutpoint. (A) Receiver Operator Curve of samples tested on both PRNT and Luminex. **(B)** Comparison of the sensitivity (blue) and sensitivity (red) as a function of cutpoint (threshold).

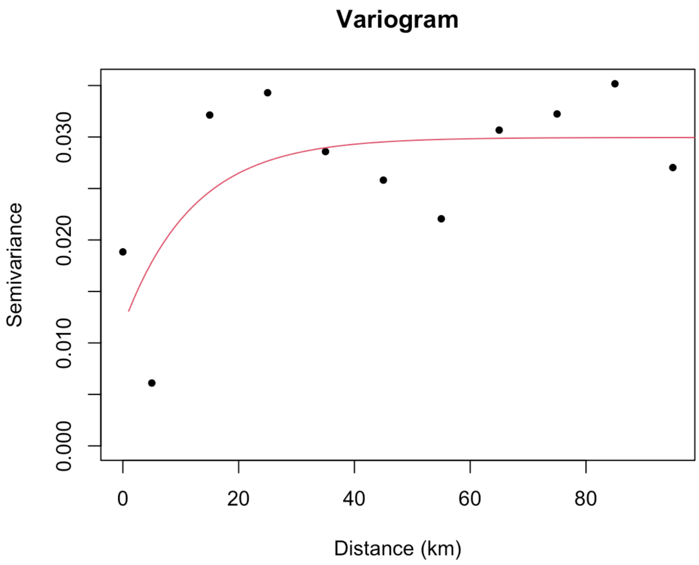

Figure S4: Semivariance in seropositivity across communities with a fitted exponential model.

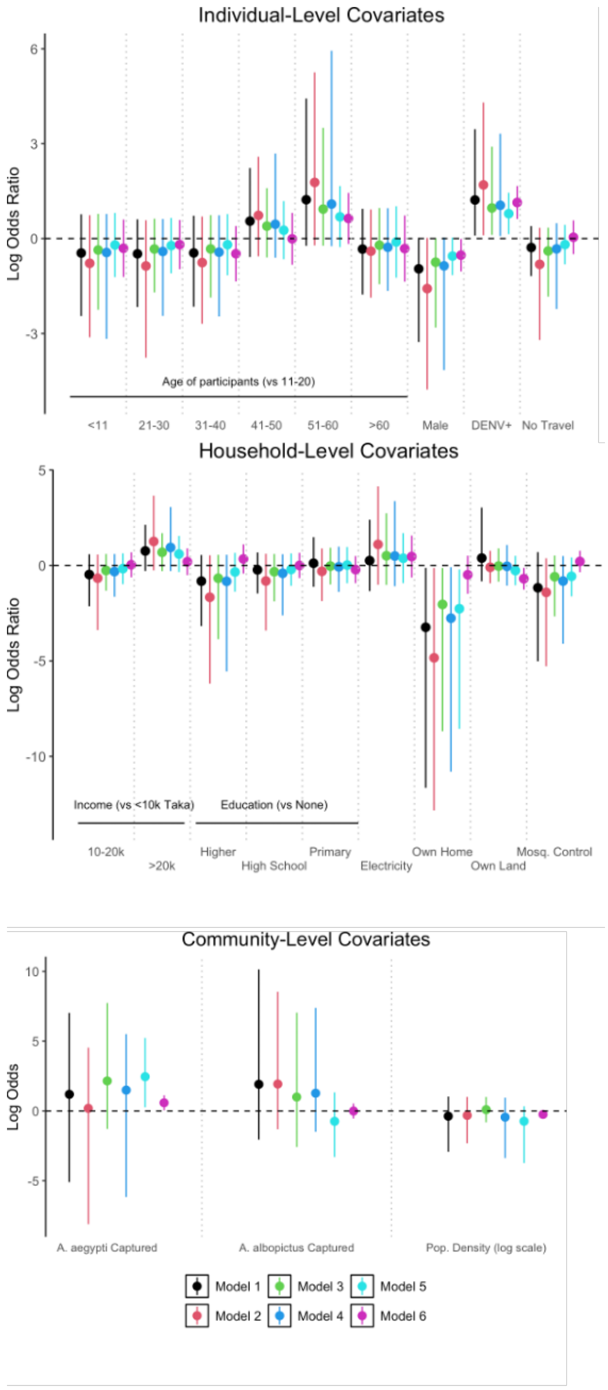

Figure S5: Difference in multivariable coefficient estimates. Run using logistic regression with a Matérn spatial correlation structure, random community intercept and a random household intercept (Model 1), a Matérn spatial correlation structure and random household intercept only (Model 2) a Matérn spatial correlation structure and random community intercept only (Model 3), a Matérn spatial correlation structure only (Model 4), random household and community intercepts only (Model 5), and random household but fixed community intercepts only (Model 6).

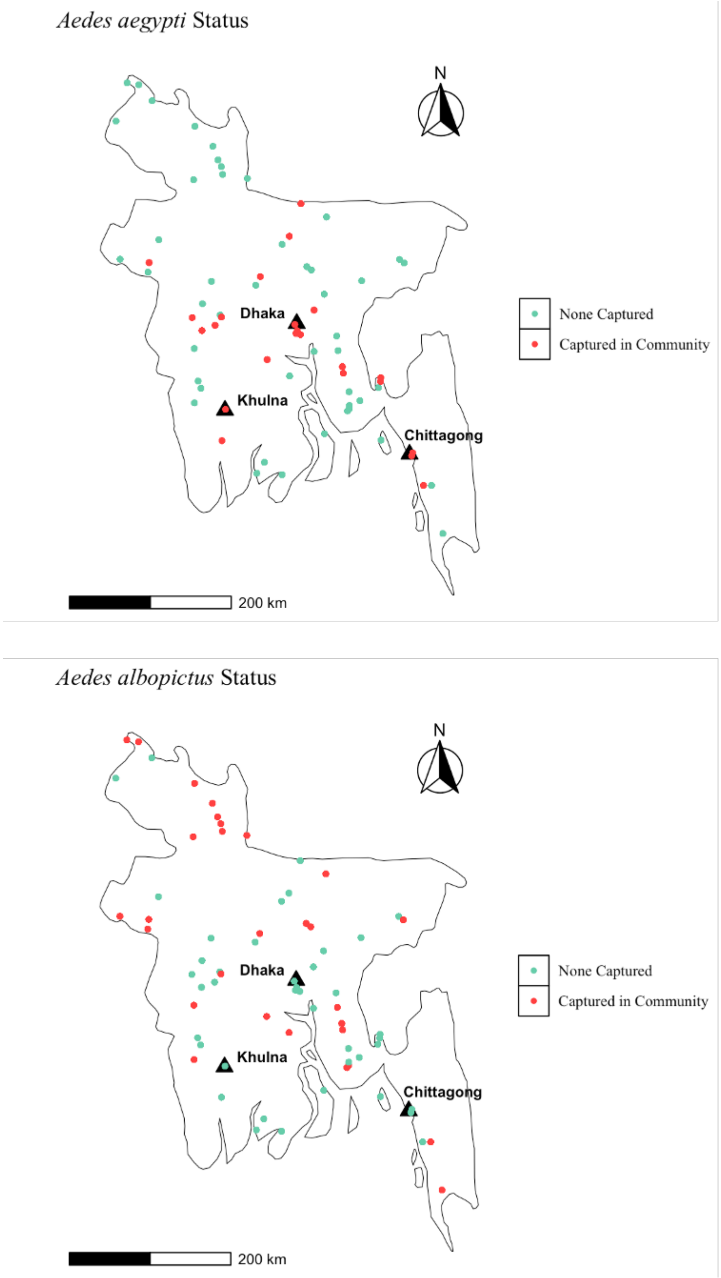

Figure S6: Presence of *A. aegypti* and *A. albopictus* in communities. The black triangles represent the three main cities in Bangladesh.

Table S1

Model	WAIC
1 (Spatial correlation structure, random community household intercepts)	1060.78
2 (Spatial correlation structure and random household intercept)	1644.85
3 (Spatial correlation structure and random community intercept)	894.79
4 (Spatial correlation structure)	8903.44
5 (Community and household random intercepts)	794.79
6 (Random household and fixed community intercepts)	656.32

Table S1: Model comparison using widely applicable/Watanabe-Akaike information criterion.
